# Two particle-picking procedures for filamentous proteins: *SPHIRE-crYOLO* filament mode and *SPHIRE-STRIPER*

**DOI:** 10.1107/S2059798320007342

**Published:** 2020-06-17

**Authors:** Thorsten Wagner, Luca Lusnig, Sabrina Pospich, Markus Stabrin, Fabian Schönfeld, Stefan Raunser

**Affiliations:** a Department of Structural Biochemistry, Max Planck Institute of Molecular Physiology, Otto-Hahn-Strasse 11, 44227 Dortmund, Germany

**Keywords:** *SPHIRE-crYOLO*, *SPHIRE-STRIPER*, cryo-EM, particle picking, filaments, deep learning

## Abstract

Structure determination of filamentous molecular complexes involves the selection of filaments from cryo-EM micrographs. The automatic selection of helical specimens is particularly difficult, and thus many challenging samples with issues such as contamination or aggregation are still manually picked. Here, two approaches for selecting filamentous complexes are presented: one uses a trained deep neural network to identify the filaments and is integrated in *SPHIRE-crYOLO*, while the other, called *SPHIRE-STRIPER*, is based on a classical line-detection approach. The advantage of the *crYOLO*-based procedure is that it performs accurately on very challenging data sets and selects filaments with high accuracy. Although *STRIPER* is less precise, the user benefits from less intervention, since in contrast to *crYOLO*, *STRIPER* does not require training. The performance of both procedures on Tobacco mosaic virus and filamentous F-actin data sets is described to demonstrate the robustness of each method.

## Abstract 2

Two approaches for the selection of filaments from cryo-EM micrographs are described.

## 1. Introduction   

Many proteins of biological and medical relevance form filaments. Prominent examples are cytoskeletal proteins such as microtubules and actin, which are essential for many cellular functions, including muscle contraction and cargo transport (Pospich & Raunser, 2018 ▸). Further examples are amyloid and tau fibrils, which are involved in neurodegenerative diseases and have recently been the focus of many structural studies (Fitzpatrick *et al.*, 2017 ▸; Pospich & Raunser, 2017 ▸). As filaments are, in general, reluctant to crystallize, cryo-EM is the method of choice to study their structure, as illustrated by the increasing number of deposited helical structures (https://www.ebi.ac.uk/pdbe/emdb/statistics_emmethod.html).

The determination of protein structures using single-particle cryo-EM requires the selection of thousands of particles within micrographs. For single particles, various methods have been developed to automate this task (Voss *et al.*, 2009 ▸; Scheres, 2015 ▸; Huang & Penczek, 2004 ▸; Nicholson & Glaeser, 2001 ▸; Wagner *et al.*, 2019 ▸; Bepler *et al.*, 2019 ▸; Tegunov & Cramer, 2019 ▸; Wang *et al.*, 2016 ▸; Zhu *et al.*, 2017 ▸). In particular, the introduction of deep-learning-based procedures have dramatically reduced the false-positive rates of picking and have made the automatic picking of particles the standard in single-particle cryo-EM (Wagner *et al.*, 2019 ▸; Bepler *et al.*, 2019 ▸; Tegunov & Cramer, 2019 ▸; Wang *et al.*, 2016 ▸; Zhu *et al.*, 2017 ▸).

However, the picking of filaments is more challenging because of the line-like structure of the specimens. It is especially difficult to omit filament crossings and overlaps. Although procedures have been introduced that allow the automated picking of helical samples (Huber *et al.*, 2018 ▸; He & Scheres, 2017 ▸), a deep-learning-based helical specimen picker is missing.

Here, we present a new deep-learning filament-picking procedure implemented in our single-particle selection tool *SPHIRE-crYOLO* (Wagner *et al.*, 2019 ▸). *CrYOLO* is based on a convolutional neural network (CNN) and the ‘you only look once’ (YOLO) approach (Redmon & Farhadi, 2017 ▸). CNNs are deep-learning network architectures that have become prominent in machine learning during the last ten years. Today, CNNs are the state-of-the-art choice for image classification and object localization.

A traditional CNN-based classifier trained on a set of positive (*e.g.* particles) and negative (*e.g.* contamination or background) examples can be turned into an object-detection system by using a sliding window. This moving window slides over the input image, crops out small regions from it and then classifies these regions as either containing a particle or not. This allows the localization of particles within micrographs. However, this approach has very limited spatial contextual information and is slowed down by a high computational overhead.

The ‘you only look once’ (YOLO) framework described by Redmon *et al.* (2016 ▸) is, among others (Mittal *et al.*, 2019 ▸), an alternative to the sliding-window approach. Instead of many small cropped-out regions, the whole micrograph goes through the network in a single pass. Internally, the image is divided into a grid, where each grid cell is responsible for predicting a single box. The confidence that a grid cell actually contains a particle, the relative box position inside the grid cell, and the width and height of a box are estimated by each individual grid cell. This approach reduces the computational overhead and makes YOLO fast, while retaining its accuracy. Moreover, because the network sees the complete image at once, it is also able to learn about the spatial context of the particles. These properties make the generic YOLO framework an excellent basis for particle picking in *crYOLO*. *CrYOLO* enables the automated picking of particles within low signal-to-noise ratio cryo-EM micrographs with minimal human supervision or intervention.

In the new filament mode, *crYOLO* places boxes on the filaments after training on several manually labeled micrographs. An extra post-processing step uses these boxes as support points to trace the actual filaments. As *crYOLO* always takes the larger context into account, it is able to skip dense filament regions or broken areas of filaments without the need for additional, user-selected thresholds. This enables *crYOLO* to identify filaments on previously unseen micrographs with an accuracy that is similar to manual picking.

In addition, we present *STRIPER* as an alternative to the filament mode in *crYOLO*. *STRIPER* enhances linear structures within in an image using oriented Gaussian smoothing kernels and then applies a line-detecting algorithm. Potential crossings are detected by the same algorithm and can be skipped.

Both methods have different hardware requirements, are based on different detection principles and require different starting conditions. For example, *crYOLO *needs a GPU to run, uses a CNN for detection and requires manual training. *STRIPER*, in contrast, runs on a CPU, uses classic line detection and only requires a few parameter adjustments to run. Given this diversity, we believe that the procedures complement each other and thus are both very useful for the cryo-EM community.

## 2. Materials and methods   

### 2.1. Oriented Gaussian filtering for feature extraction   

**Figure 1:**
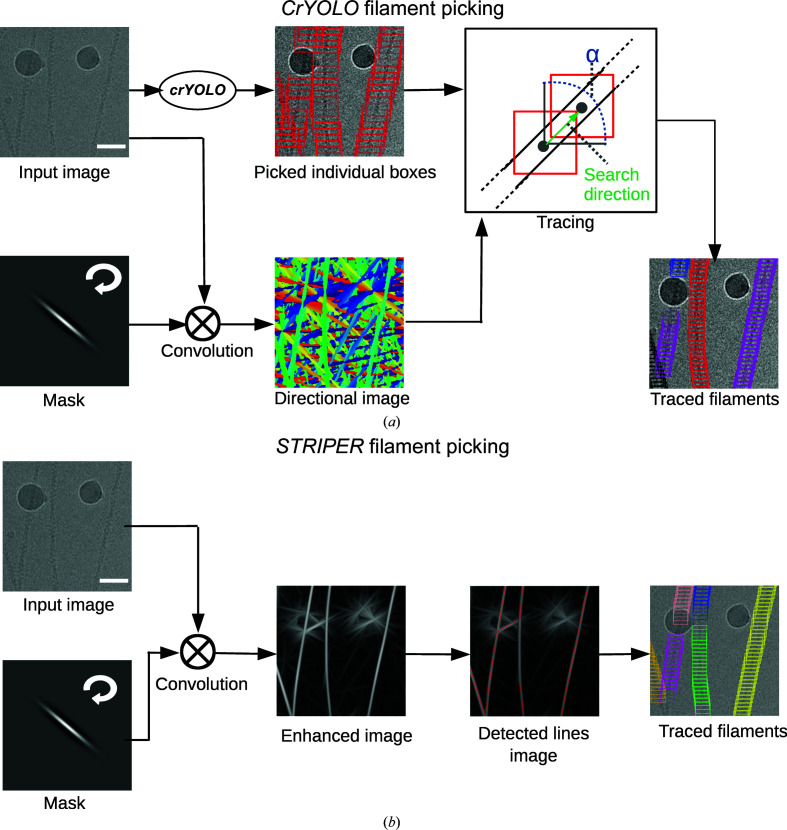
Filament picking with *crYOLO* and *STRIPER*. (*a*) *CrYOLO*. The input image is convolved multiple times with rotated versions of the convolutional mask. Each pixel in the directional image is color-coded to indicate the direction of the mask with the strongest response at its coordinates. During tracing, a box is randomly chosen and the search direction is determined by the directional image. The search angle α is set to 120°. The search radius is set proportional to the box size. Finally, given the traced boxes, the filament boxes are generated using a pre-set distance. (*b*) *STRIPER* convolves the input image with the same mask as *crYOLO*. An enhanced image is created by setting each pixel value to the strongest response of the rotated convolutional filters. The enhanced lines are then detected by a line-tracing algorithm (Steger, 1998 ▸; Wagner & Hiner, 2017 ▸). After tracing, crossing points are removed and the boxes are placed along the detected lines at a user-defined distance. Scale bars represent 50 nm.

An oriented Gaussian smoothing kernel can be used to extract direction-dependent information from an image and/or enhance specific directional features of an image. Here, we use an oriented Gaussian smoothing kernel to extract line features. The second derivative in the *y* direction *M*(*x*, *y*) of a smoothing kernel is given by
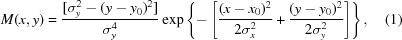
where σ_*x*_ and σ_*y*_ are the spread in the *x* and *y* directions, respectively, and *x*_0_ and *y*_0_ denote the center of the kernel. The spread σ_*y*_ determines the amount of averaging in the *y* direction and the spread σ_*x*_ is proportional to the width of the line structure it enhances (see the mask in Fig. 1 ▸).

To enhance lines with a specific orientation θ, *M* needs to be rotated by θ. Let *I*(*x*, *z*) be our input image; the oriented filter response *R*(*x*, *y*) then enhances structures that are oriented in the direction θ, 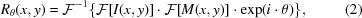
where 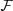 denotes the Fourier transform. To enhance all line structures with arbitrary orientation, we calculate
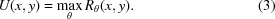
To extract the dominating direction at every position in an image, we calculate
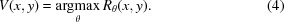

*V* is used in the *crYOLO* tracing method and *U* is used in *STRIPER* for running the line-detection algorithm.

### 2.2. Steger line detection   

The *STRIPER* ridge-detection algorithm is based on Steger (1998 ▸). It identifies any lines present within the image through differential geometric properties. More precisely, the algorithm is divided into four steps.

(1) Pre-processing based on the approach of Koller *et al.* (1995 ▸), where the image is filtered with the derivative of a Gaussian smoothing kernel. The resulting image features a series of mathematical properties (Koller *et al.*, 1995 ▸) that allow the algorithm to detect lines of arbitrary width.
(2) Detect all the pixels on an identified line segment (‘line points’). For each line point a strength *s* is calculated which is a measure of belonging to a particular line. In a greyscale image, pixels that are not line points are assigned an *s* value of 0.0, while line points closer to the center of a line have an *s* value of up to 1.0.
(3) Connect line points to form the actual lines and identify line crossings. The list of line points *L* is first reduced by removing any line points with an *s* value lower than a user-defined threshold.The procedure for building a generic line *o* is a generalization of a hysteresis threshold operation (Canny, 1986 ▸).
  (*a*) Select the line point *p* with the highest *s* value as the starting point of a new line *o*.
  (*b*) From the surrounding pixels of *p*, select the one with highest *s* value that is not already part of *o* and add it to *o*.
  (*c*) Repeat (*b*) until (i) no valid line point is found, thus indicating the end of the line, or (ii) the selected line point is already part of a different line. Mark this point as a junction and split the line in two.New lines are created until all points in *L* have been visited once (Steger, 1998 ▸).
(4) Determine the line width. Since the edges of a thick line are lines themselves, they are identified in a similar way as above by using a different filter.

Steger uses a generic line model defined by the *s* values of each pixel in step (2), and the computed line widths, to improve the position of the estimated line (Steger, 1998 ▸).

### 2.3. *CrYOLO*   

*CrYOLO* is a particle-picking procedure based on the YOLO framework (Redmon & Farhadi, 2017 ▸). For a technical description of *crYOLO*, we refer the reader to our original publication (Wagner *et al.*, 2019 ▸).

### 2.4. Evaluation procedures   

For the evaluation of the proposed procedures, we used the common metrics of recall and precision. The recall score measures the ability of the classifier to detect positive examples, and the precision score measures the ability of the classifier to not label a true negative as a true positive. Both measurements are commonly used for binary classification tasks. To calculate the precision and recall for the user-selected filaments *T*_u_ and the results *T*_p_ given by *crYOLO* or *STRIPER*, we performed the following.

(i) We transformed traced filaments of *T*_u_ into a binary image *B*_u_ by setting all pixels along the filament and within a local radius of *f*_w_/3 to the value 1. The same is performed for *T*_p_, which results in the binary image *B*_p_.
(ii) We calculated a difference image *D* by *D* = *B*_u_ − *B*_p_. Here, we define *D*_1_ as a binary image in which all pixels in *D* are set to 0 except the positive pixels and *D*_−1_ as a binary image in which all pixels in *D* are set to 0 except the pixels with negative values.
(iii) The false-negative pixels (FN) and the false-positive pixels (FP) are then given by
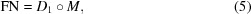


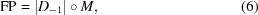
where *M* is a mask of ones of size *f*_w_ × *f*_w_ and ○ is the morphological opening operator. The true positive pixels are then given by
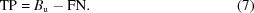
Finally, the precision and recall are defined as
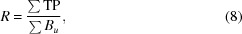


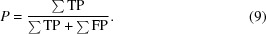

To calculate these statistics, we ignored both picks on the border of the image and the start and end positions of each filament, as they are connected with high uncertainty during manual selection.

## 3. Results and discussion   

### 3.1. *CrYOLO* filament mode   

Since we have only recently introduced the *crYOLO* filament mode, a generalized model is not yet available. Therefore, *crYOLO* requires several filaments to be labeled manually in order to properly train the model. We used the *e*2*helixboxer* program provided by *EMAN*2 (Tang *et al.*, 2007 ▸) for manual selection, but any other program that allows the manual picking of filaments can be applied. The number of micrographs that need to be manually annotated may vary depending on the filament density, orientation and background variations. Also, a project with many aggregates might increase the number of training images needed to obtain a good working model.

The training of *crYOLO* works as described for single-particle projects (Wagner *et al.*, 2019 ▸). The network is trained on the manually labeled micrographs, while a small subset of those micrographs is used for validation. After each round of training, *crYOLO* measures the success of picking on the validation micrographs and stops the training when the validation performance no longer increases. After training is complete, *crYOLO* goes through the data set and places boxes on filaments. The boxes have no defined distance to each other and no information about the filament to which they belong. As a special requirement for helical specimens, the filament mode in *crYOLO* allows more overlap of boxes during picking. The originally determined positions of the boxes on the filaments are merely used as support points for placing new boxes with a user-defined distance. During this post-processing step, an oriented convolutional mask filter is used to estimate the direction of every filament.

The mask contains the second derivative of an oriented Gaussian smoothing kernel (see equation 1). Given the user-selected filament width *f*_w_ in pixels and a default mask width *m*_w_ of 100 pixels, then σ_*x*_ and σ_*y*_ are defined using the ‘full width at half maximum’ criterion:
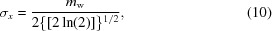


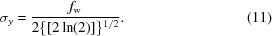
The kernel is rotated and each rotated version is convolved with the input image. For each pixel in the input image, the rotational angle of the convolutional mask that gives the maximum response is determined (see equation 4), which when evaluated for all pixels gives the directional map (Fig. 1 ▸ *a*).

Next, a box is chosen randomly on the filament and the direction of the filament is evaluated by the directional map. The next box is searched for within a radius proportional to the box size; the search is restricted by an angle α around the estimated direction of the filament. If a box can be found, the search is continued. If not, another as yet untraced box is selected and the search is repeated. In the case where the search finds a previously traced box, both filament segments will be merged into a single filament if their directions are comparable. After the tracing has been performed, new boxes are generated based on the user-defined distance. The final boxes are saved in STAR and *EMAN* helical box format.

The filament mode of *crYOLO* has already been successfully applied to solvie the structures of F-actin in complex with drug-like toxins (Pospich *et al.*, 2020 ▸) and with LifeAct (Belyy *et al.*, 2020 ▸).

### 3.2. *STRIPER*   

Instead of using a trained deep neural network to identify the boxes along the filaments, the *STRIPER* filament-picking procedure is based on a classical line-detection approach (Fig. 1 ▸ *b*). Cryo-EM images typically have a very low signal-to-noise ratio, which is problematic for line-detection algorithms. We therefore included a line-enhancing pre-processing step in *STRIPER*. In this step, the filaments are enhanced using the same oriented Gaussian smoothing kernel as described above for *crYOLO*. The width of the mask is configured by the user and should be set to the filament width. The lines in the enhanced image (see equation 3) are then extracted by the ridge-detection algorithm of Steger (Steger, 1998 ▸; Wagner & Hiner, 2017 ▸).

To run the *STRIPER* filament procedure, four parameters need to be provided: (i) the filament width in pixels, which can easily be measured, (ii) the mask width in pixels, which is set to 100 by default and only has to be changed for very flexible filaments (both parameters are used for the creation of the enhanced line image), and (iii) the upper and (iv) the lower threshold used as hysteresis thresholds for Steger’s line-detection algorithm (see Section 2). Since the latter values are difficult to guess, *STRIPER* provides an optimization algorithm to estimate these parameters. For this, the user has to manually select the filaments in two to three micrographs. While fixing the lower threshold to a value of 0, a simple grid search will then find the best upper threshold to detect as many of the annotated filaments as possible. Finally, the lower threshold is increased stepwise to remove false-positive detections. After extracting the lines, *STRIPER* splits them at crossing points, and boxes are placed along the lines with a user-defined distance.

By defining a minimum length for detected filaments, *STRIPER* can remove short line-like contaminations that are detected as false positives. Moreover, *STRIPER* allows the user to provide a binary mask for each micrograph. This mask divides an image into valid picking regions and regions with carbon or contamination. This masking option is especially useful as deep-learning-based carbon and contamination detection have recently become available (Tegunov & Cramer, 2019 ▸; Sanchez-Garcia *et al.*, 2020 ▸). These programs determine valid and nonvalid regions with high accuracy and create binary masks, which then can be directly used in *STRIPER* to remove false-positive selections.

*STRIPER* has already been successfully applied to solve the structures of toxin-stabilized F-actin (Pospich *et al.*, 2020 ▸) and of F-actin in the ADP-P_i_ state (Merino *et al.*, 2018 ▸).

### 3.3. Training and configuration   

To test both procedures, we used the publicly available Tobacco mosaic virus (TMV) data set (EMPIAR 10022; Fromm *et al.*, 2015 ▸) and one of our in-house F-actin data sets (Belyy *et al.*, 2020 ▸). The TMV data set has two difficulties: Firstly, several filaments are localized in very close proximity on the grid and should ideally not be selected. Secondly, the TMVs contain interruptions or discontinuities in their structure, which should be excluded from selection. The challenges of the F-actin data set are that the filaments often cross each other and that large carbon areas are covered with F-actin. In contrast to the TMV data set, the F-actin images contain carbon edges, which is especially demanding for the selection process, since they appear as line-like structures.

To train *crYOLO* on both data sets, we used manually labeled filaments on several micrographs. For F-actin, we selected 275 filaments on 24 micrographs. For TMV, we used the manually traced filaments that were provided with the EMPIAR data set and selected a subset of 425 labeled filaments from 44 micrographs. For both data sets we included images with a broad defocus range. This is important as otherwise the detection quality on a low-defocus image might be low. We roughly estimated the width of TMV and actin filaments on the images and used these values for processing (∼200 Å for TMV and ∼80 Å for F-actin). Since the distance of the boxes is not relevant for the selection of the filaments, we used a standard box distance of 20 pixels for both data sets. When processing the data further for structural investigations this value should be adjusted, taking the helical rise of the filament into account. The picking threshold in *crYOLO* was set to the default value of 0.3 for both data sets. For evaluation, 20% of the labeled micrographs were not used during training.

In *STRIPER*, several processes that are automatically performed in *crYOLO*, such as binning, normalization and filtering, have to be performed manually. Thus, to test *STRIPER* we binned the TMV and F-actin images by a factor of four, low-pass filtered them with an absolute cutoff frequency of 0.1 and normalized them by subtracting the mean and dividing by the standard deviation. All pixel values greater than 3 or lower than −3 were saturated.

The contrast of an image has a strong influence on the line-detection algorithm used in *STRIPER*. Since the contrast depends very much on the defocus at which the images have been taken, we manually labeled filaments in one micrograph with high defocus and one micrograph with low defocus to determine the hysteresis thresholds. We then applied the internal grid-optimization routine to find the best set of selection thresholds (upper and lower thresholds of 0.77 and 0.29 for F-actin, and 0.2 and 0.1 for TMV, respectively).

For the evaluation of the precision and recall for *crYOLO* filament mode and *STRIPER*, we used the same micrographs.

### 3.4. Evaluation on test data sets   

**Figure 2:**
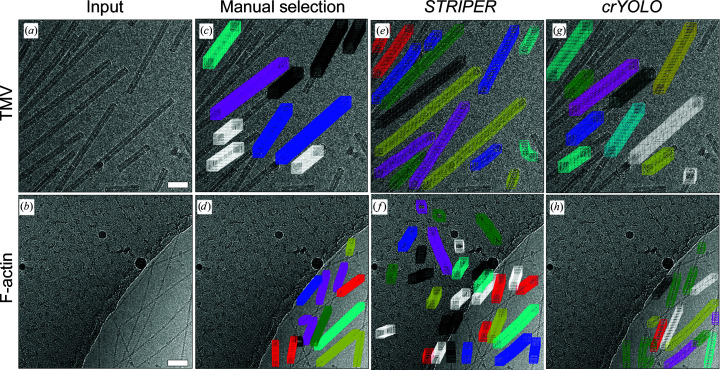
*CrYOLO* and *STRIPER* evaluated on micrographs with F-actin and TMV. (*a*, *b*) Input images that were not used during training (*crYOLO*) or parameter optimization (*STRIPER*). (*c*, *d*) Manually selected filaments. (*e*–*h*) Automatically selected filaments by *STRIPER* (*e*, *f*) or *crYOLO* (*g*, *h*). Scale bars represent 50 nm.

When we applied *crYOLO* to the TMV data set, the recall and precision were 0.98 and 0.84, respectively. The automatic picking of filaments resulted in a selection of filaments that was comparable to the manually picked data sets. Filaments that touched each other were skipped and discontinuities in the filaments, as are typical for this TMV data set (Supplementary Fig. S1), were mostly omitted (Fig. 2 ▸ *g*). In contrast, *STRIPER* identified and picked almost all filaments on the micrographs (Fig. 2 ▸ *e*). This led to a high recall (1.0) but, since *STRIPER* also selected filaments that sit very close to each other and contain discontinuities, the precision of only 0.52 was quite low.

For F-actin filaments, *crYOLO* achieved a recall of 0.95 and a precision of 0.83. It skipped most of the filament crossings and did not select the carbon edge or filaments on the carbon (Fig. 2 ▸ *h*). *STRIPER* also identified most of the filaments and skipped their crossings (Fig. 2 ▸ *f*). However, it also picked the carbon edge as well as filaments and line-like contaminations on the carbon. The recall was 0.81 and the precision was 0.51. As *STRIPER* supports binary masks, we masked out the carbon area using the *MicrographCleaner* tool (Sanchez-Garcia *et al.*, 2020 ▸) and repeated the selection (Supplementary Fig. S2). The masking led to an increase in the precision of *STRIPER* to 0.73, while the recall remained at 0.81.

We further assessed the quality of the selected filaments by 2D classification in *SPHIRE* (Moriya *et al.*, 2017 ▸; Yang *et al.*, 2012 ▸). We then applied *Cinderella* (Wagner, 2020 ▸), a deep-learning-based tool that is trained to identify high-quality particle classes, and used the percentage of rejected classes as an indication of the quality of the picking procedure.

**Figure 3:**
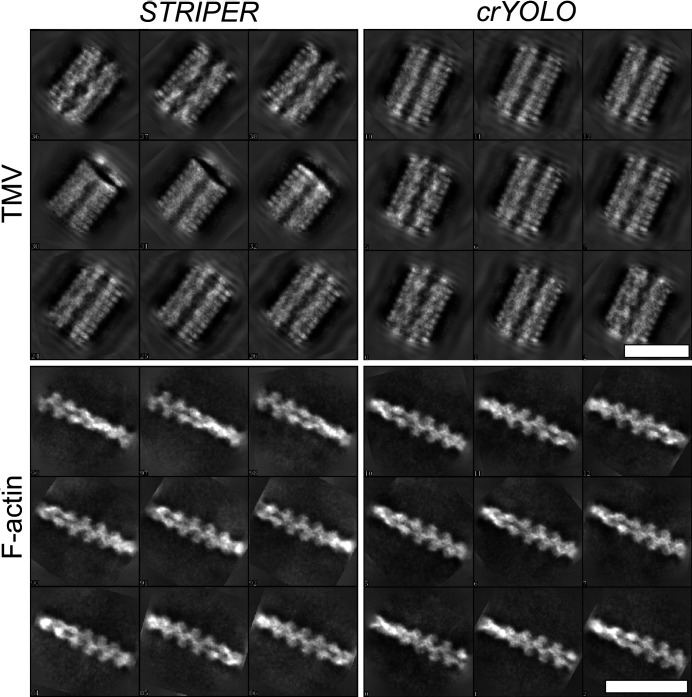
Example class averages calculated in *SPHIRE*. F-actin and TMV were picked by *crYOLO* filament mode or *STRIPER*. The respective numbers of class averages are listed in Table 1 ▸. Scale bars represent 25 nm.

The particles picked by both selection procedures resulted in many high-quality class averages (Fig. 3 ▸). Almost all classes calculated for the filaments identified by *crYOLO* were accepted by *Cinderella* (Table 1 ▸, Supplementary Figs. S3 and S4), demonstrating that *crYOLO* indeed did not pick background, contamination or carbon edges.

For particle stacks selected with *STRIPER*, almost all class averages were accepted by *Cinderella* in the case of TMV (Supplementary Fig. S5). The number of selected particles is much higher than for *crYOLO*, because *STRIPER* also picked TMVs with discontinuities and filaments that were in close proximity to their neighbors. As expected, 37% of the classes mainly contained false positives and were rejected in the case of F-actin (Supplementary Fig. S6). Applying a mask to exclude the carbon and carbon edges solved this problem and almost all classes were accepted by *Cinderella* (Table 1 ▸). However, the total number of accepted classes is much lower compared with the classes obtained from particles selected by the *crYOLO* filament mode (Table 1 ▸).

### 3.5. Computational efficiency   

To determine the speed, we picked 203 F-actin micrographs (4096 × 4096) using an Intel Xeon Gold 5122 CPU and an Nvidia Titan V GPU (32 GB RAM). On average, *crYOLO* picked a micrograph in 7 s (including filtering and filament post-processing), while *STRIPER* required 0.8 s (without filtering). In both cases the algorithms presented in this paper provide highly efficient picking methods that allow users to pick large data sets in short amounts of time.

## 4. Conclusions   

Particle picking is a crucial step in the cryo-EM processing pipeline. Picking of helical specimens is particularly challenging, as crossings, overlaps and filaments that are in too close proximity to each other need to be omitted. This challenge is well illustrated by the data sets used as examples in this work. Here, we present two picking procedures that allow accurate picking of filaments even for complex data sets, as illustrated for TMV and F-actin in this work. One of them is based on the deep-learning particle-picking procedure *crYOLO* and the other, called *STRIPER*, is based on a line-detection algorithm. *CrYOLO* is to our knowledge the first deep-learning-based particle-picking procedure which supports filaments. Through this approach, *crYOLO* learns the spatial context of filaments, enabling it to omit carbon edges and filaments that are too dense or broken, thereby accurately reproducing the accuracy of manual picking. As no general model for filaments is yet available, *crYOLO* requires training on manually selected micrographs. In contrast, *STRIPER* only requires four parameters, which can be quickly determined by the integrated optimization procedure. While this makes *STRIPER* a very fast, easily accessible picker, it comes at the cost of reduced precision compared with *crYOLO*. However, we also showed that the usage of a binary mask significantly improves the precision of *STRIPER*, resulting in high-quality 2D classes. Both picking procedures produce box files that are compatible with the majority of cryo-EM processing software and come with standard hardware requirements. Considering the performance and accessibility, we believe that both procedures are seminal contributions to the cryo-EM field.

## 5. Code availability   

The filament mode of *crYOLO* has been available since *crYOLO* version 1.3. *CrYOLO* itself is free for academic use and can be downloaded together with the source code at http://sphire.mpg.de/. *crYOLO* is implemented in Python and based on *Keras* 2.2.5 (https://keras.io/) using *Tensorflow* 1.10.1 (Abadi *et al.*, 2016 ▸) as the backend. The version of *STRIPER* used in this paper was first implemented as an *ImageJ* (Rueden *et al.*, 2017 ▸) plugin and is open source. The source code can be found at https://github.com/MPI-Dortmund/ij_striper. A Python implementation of *STRIPER* is currently being implemented and the alpha version can be found at https://github.com/MPI-Dortmund/striper. All data supporting the findings of this study are available from the corresponding author on reasonable request.

## Supplementary Material

Supplementary figures. DOI: 10.1107/S2059798320007342/ic5112sup1.pdf

## Figures and Tables

**Table 1:** Results of 2D classification for F-actin and TMV The number of class averages and the number of classes accepted by *Cinderella* were evaluated for F-actin and TMV for *crYOLO* filament mode, *STRIPER* and *STRIPER* with masks.

		No. of particles		No. of classes	
Data set	Procedure	Accounted for	Unaccounted for	Total	Accepted
F-actin	*crYOLO*	102262	175	1023	1019
F-actin	*STRIPER*	112118	897	1128	708
F-actin	*STRIPER* (+ masks)	69671	217	697	682
TMV	*crYOLO*	13338	154	133	133
TMV	*STRIPER*	49897	1333	503	496
