# Supplementary material for: Two particle-picking procedures for filamentous proteins: SPHIRE-crYOLO filament mode and SPHIRE-STRIPER
Source: Acta Crystallogr D Struct Biol. 2020 Jun 17;76(Pt 7):613–20. doi: 10.1107/S2059798320007342 (PMC7336381; doi:10.1107/S2059798320007342)
Supplement: Supplementary file 1 [file d-76-00613-sup1.pdf]

STRUCTURAL BIOLOGY

Volume 76 (2020)

Supporting information for article:

Two particle-picking procedures for filamentous proteins: *SPHIRE-crYOLO* filament mode and *SPHIRE-STRIPER*

Thorsten Wagner, Luca Lusning, Sabrina Pospich, Markus Stabrin, Fabian Schönfeld and Stefan Raunser

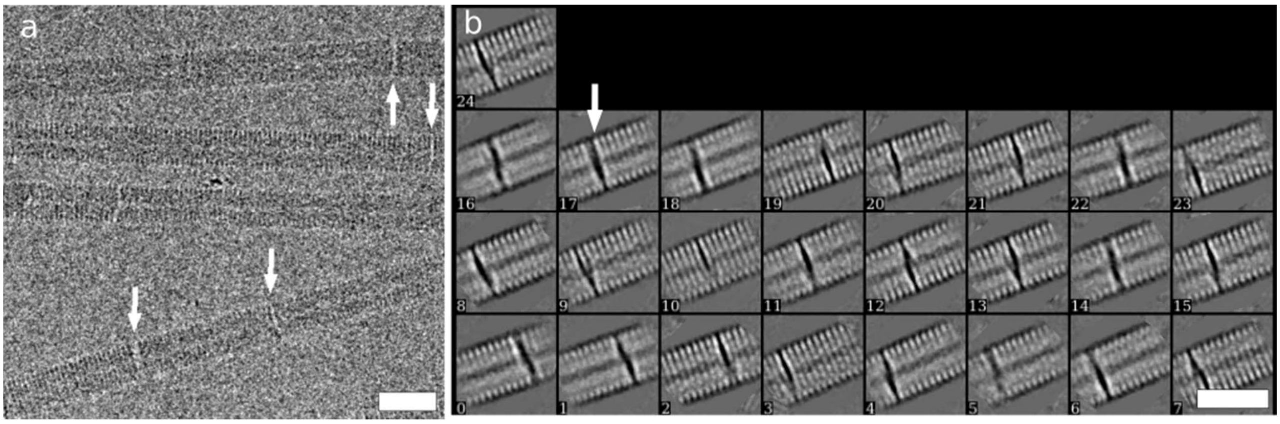

Figure S1 Discontinuities in TMV. (a,b) After picking with STRIPER (a) and 2D classification with SPHIRE discontinuities in TMV (highlighted by white arrows) also appear in 25 out of 503 classes (b). For crYOLO, only 1 class out of 133 shows a discontinuity. Scale bars, 25 nm.

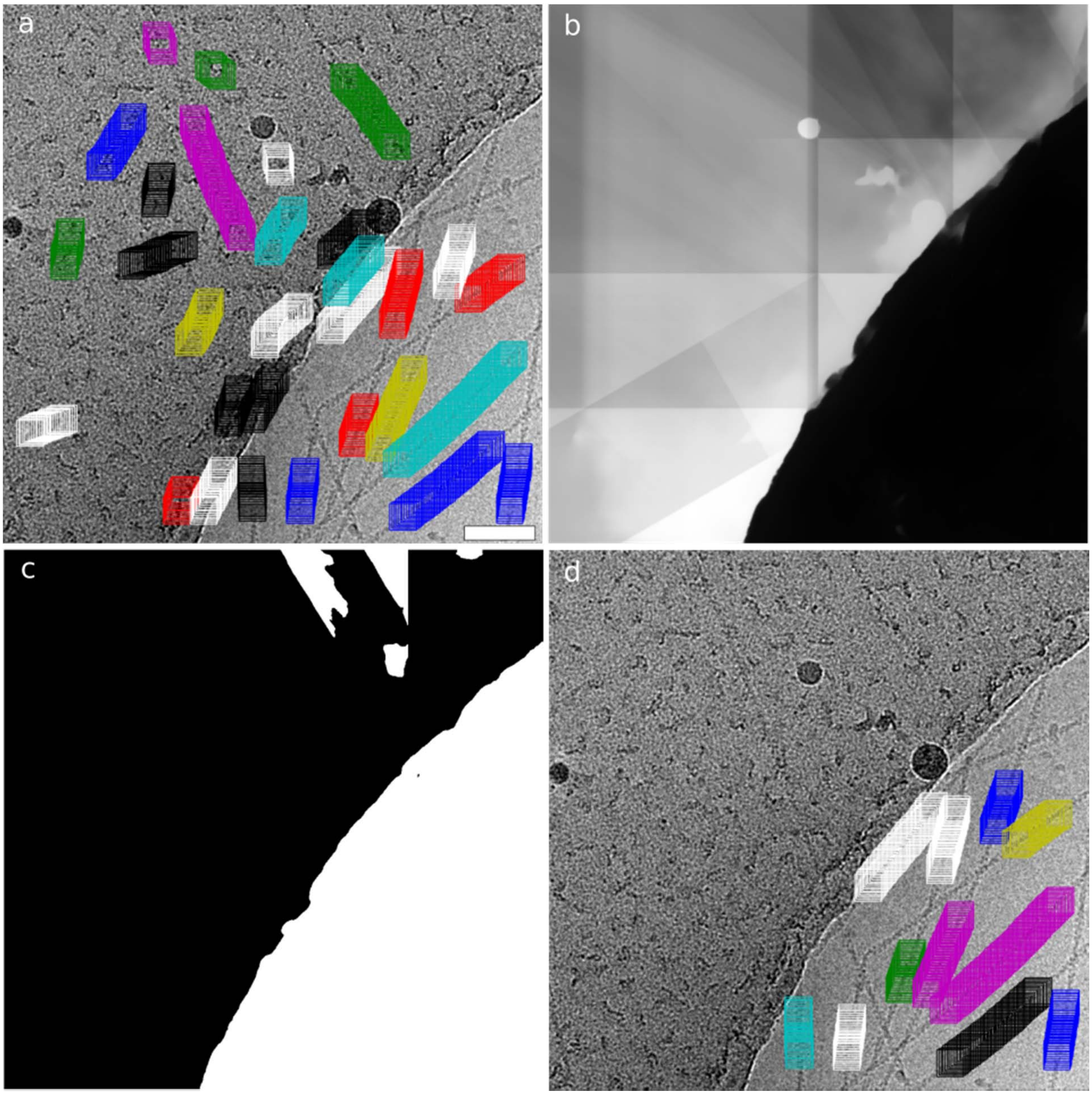

Figure S2 Elimination of false-positive STRIPER picks using a mask. (a) STRIPER tends to identify all line-like structures. This is problematic in case of line-like contamination or filaments on carbon. (b,c) Using MicrographCleaner we calculated a mask (b) and binarized it using a threshold of 0.3 (c). (d) This mask can then be used in STRIPER to remove false-positive filament picks. Scale bar, 50 nm.

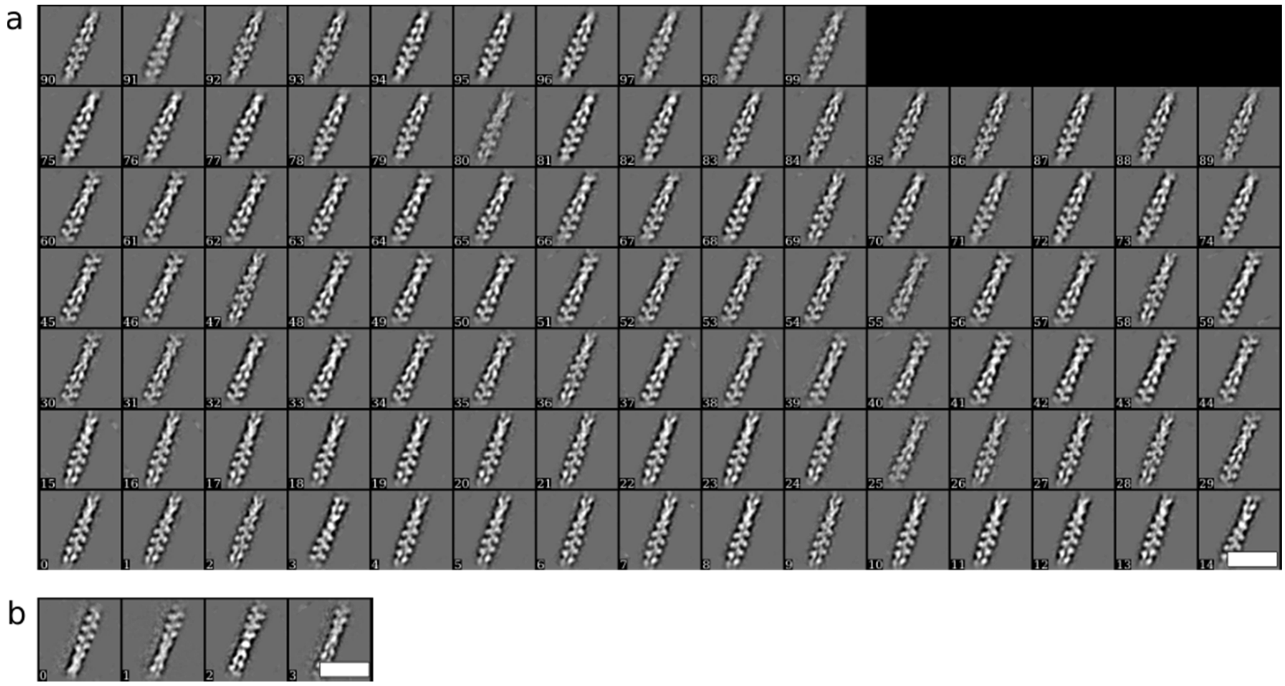

Figure S3 2D classes of F-actin accepted or rejected by Cinderella. (a) 100 out of 1023 accepted classes and (b) all rejected classes for filaments picked by crYOLO. Note that the rejected classes seem to be false-positives. Scale bars, 25 nm.

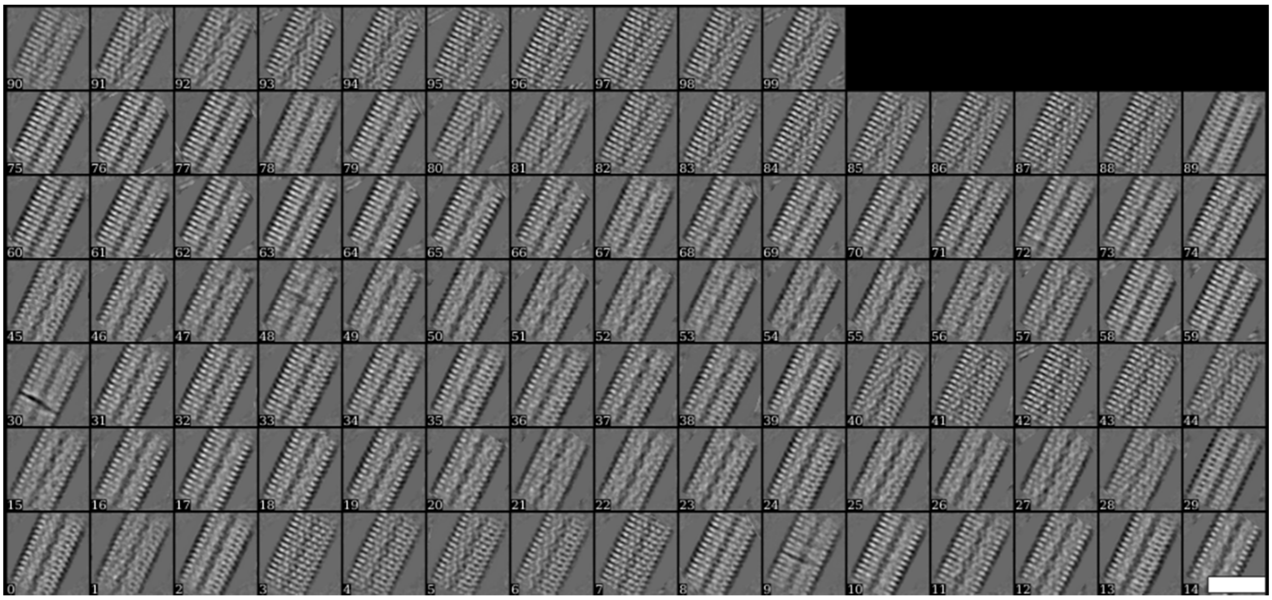

Figure S4 2D classes of TMV filaments accepted by Cinderella. 100 out of 113 classes accepted by Cinderella. Filaments were picked by crYOLO. No class was rejected by Cinderella. Scale bar, 25 nm.

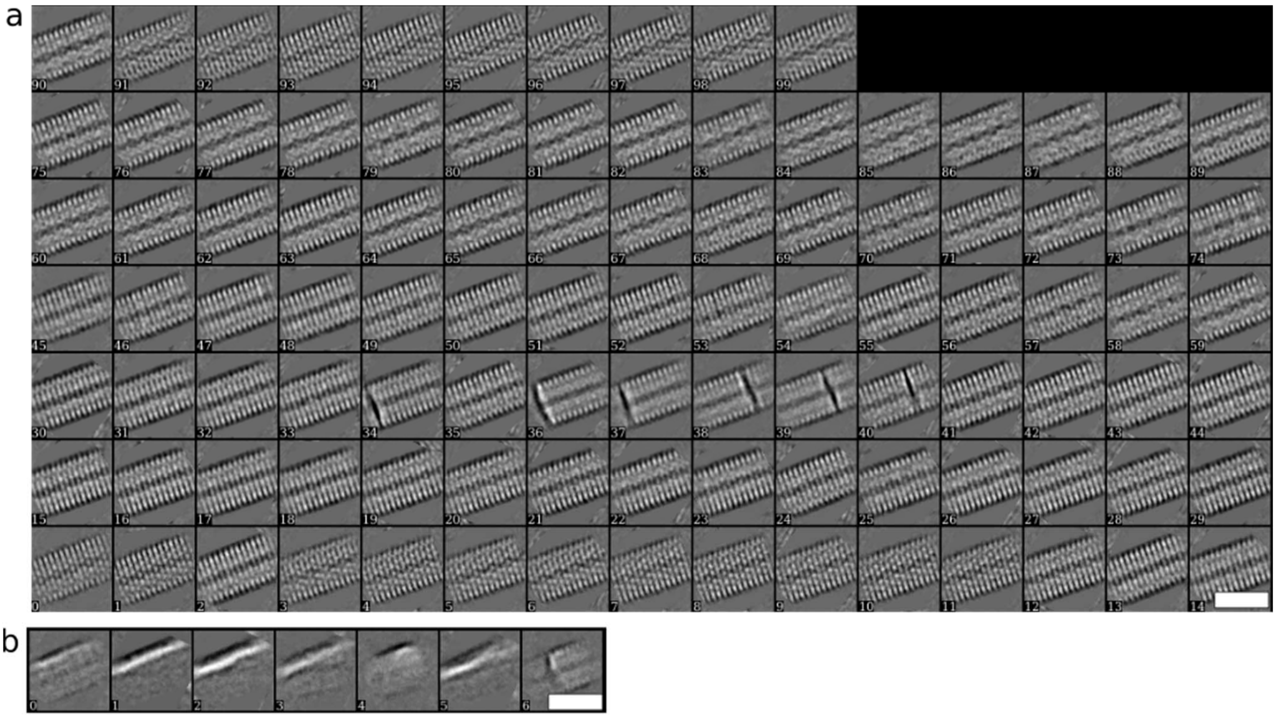

Figure S5 2D classes of TMV accepted or rejected by Cinderella. (a) 100 out of 496 accepted classes and (b) all rejected classes for filaments picked by STRIPER. Scale bars, 25 nm.

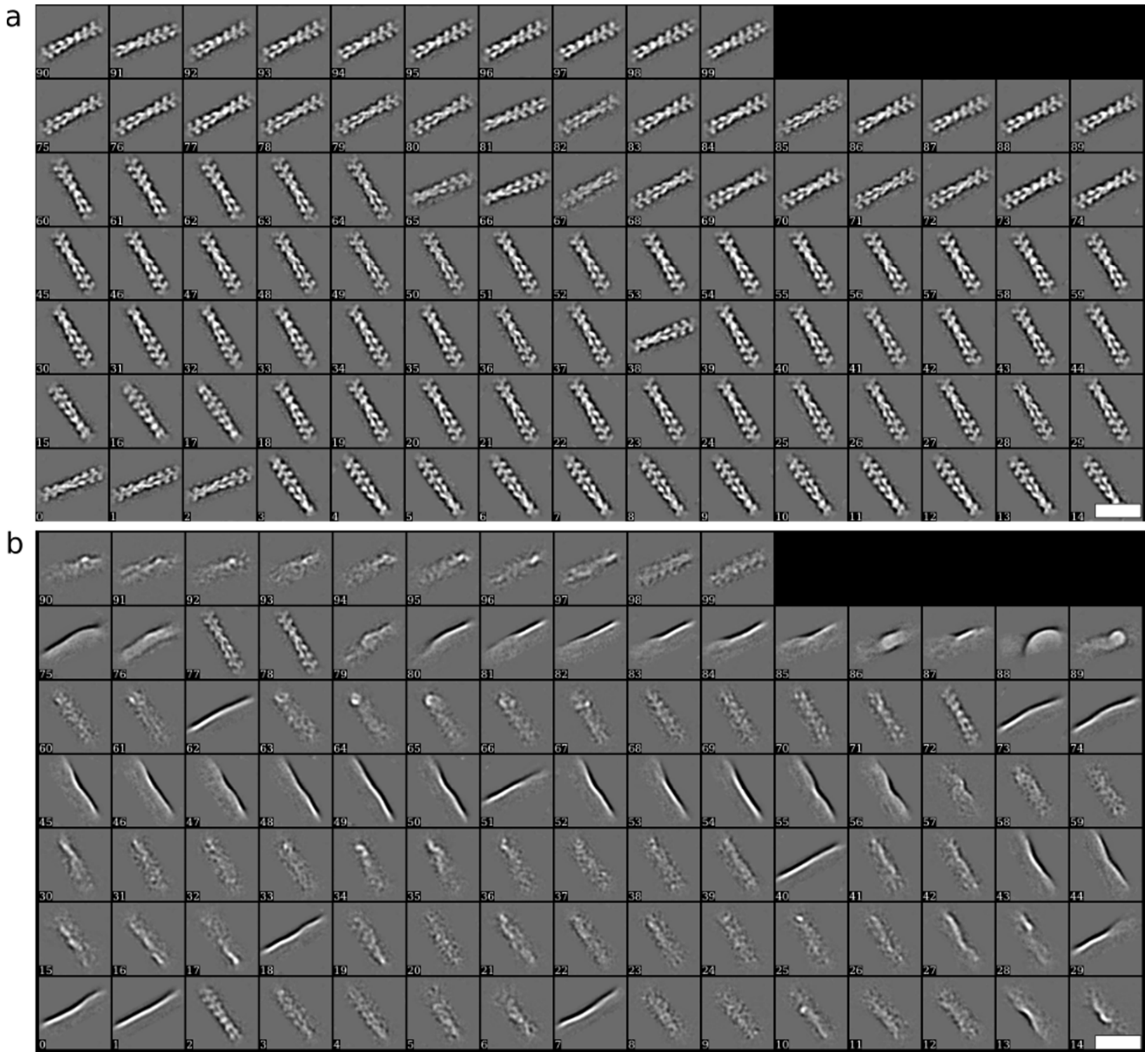

Figure S6 2D classes of F-actin filaments accepted or rejected by Cinderella. (a) 100 out of 708 accepted classes and (b) 100 out of 420 rejected classes for filaments picked by STRIPER. Scale bars, 25 nm.
